# Antidepressant Acts on Astrocytes Leading to an Increase in the Expression of Neurotrophic/Growth Factors: Differential Regulation of FGF-2 by Noradrenaline

**DOI:** 10.1371/journal.pone.0051197

**Published:** 2012-12-05

**Authors:** Naoto Kajitani, Kazue Hisaoka-Nakashima, Norimitsu Morioka, Mami Okada-Tsuchioka, Masahiro Kaneko, Miho Kasai, Chiyo Shibasaki, Yoshihiro Nakata, Minoru Takebayashi

**Affiliations:** 1 Department of Pharmacology, Graduate School of Biomedical Sciences, Hiroshima University, Hiroshima, Hiroshima, Japan; 2 Division of Psychiatry and Neuroscience, Institute for Clinical Research, National Hospital Organization (NHO) Kure Medical Center and Chugoku Cancer Center, Kure, Hiroshima, Japan; 3 Department of Psychiatry, National Hospital Organization (NHO) Kure Medical Center and Chugoku Cancer Center, Kure, Hiroshima, Japan; Chiba University Center for Forensic Mental Health, Japan

## Abstract

Recently, multiple neurotrophic/growth factors have been proposed to play an important role in the therapeutic action of antidepressants. In this study, we prepared astrocyte- and neuron-enriched cultures from the neonatal rat cortex, and examined the changes in neurotrophic/growth factor expression by antidepressant treatment using real-time PCR. Treatment with amitriptyline (a tricyclic antidepressant) significantly increased the expression of fibroblast growth factor-2 (FGF-2), brain-derived neurotrophic factor, vascular endothelial growth factor and glial cell line-derived neurotrophic factor mRNA with a different time course in astrocyte cultures, but not in neuron-enriched cultures. Only the expression of FGF-2 was higher in astrocyte cultures than in neuron-enriched cultures. We focused on the FGF-2 production in astrocytes. Several different classes of antidepressants, but not non-antidepressants, also induced FGF-2 mRNA expression. Noradrenaline (NA) is known to induce FGF-2 expression in astrocyte cultures, as with antidepressants. Therefore, we also assessed the mechanism of NA-induced FGF-2 expression, in comparison to amitriptyline. NA increased the FGF-2 mRNA expression via α1 and β-adrenergic receptors; however, the amitriptyline-induced FGF-2 mRNA expression was not mediated via these adrenergic receptors. Furthermore, the amitriptyline-induced FGF-2 mRNA expression was completely blocked by cycloheximide (an inhibitor of protein synthesis), while the NA-induced FGF-2 mRNA was not. These data suggest that the regulation of FGF-2 mRNA expression by amitriptyline was distinct from that by NA. Taken together, antidepressant-stimulated astrocytes may therefore be important mediators that produce several neurotrophic/growth factors, especially FGF-2, through a monoamine-independent and a *de novo* protein synthesis-dependent mechanism.

## Introduction

Most antidepressants increase the extracellular noradrenaline (NA) and/or serotonin (5-hydroxytryptamine; 5-HT) levels by inhibiting the reuptake of monoamine in presynaptic terminals. Although changes in extracellular monoamine levels occur soon after the drug administration, the clinical antidepressant effect develops slowly over several weeks of continuous treatment [1]. The efficacy of antidepressants cannot be solely explained by their actions on the monoaminergic neurons. The molecular and cellular adaptations that underlie the therapeutic action of antidepressants therefore still remain to be elucidated.

Over the past decade, clinical and animal studies have suggested that several neurotrophic/growth factors play important roles in the efficacy of antidepressant [1], [2], which is assumed to be associated with neuronal plasticity, such as neurogenesis and synaptogenesis [3], [4]. Clinical studies have indicated that lower levels of fibroblast growth factor-2 (FGF-2), brain-derived neurotrophic factor (BDNF), and glial cell line-derived neurotrophic factor (GDNF) in the postmortem brain or blood from patients with major depressive disorder (MDD) were attenuated by antidepressant medications [5]–[8]. Animal studies have shown that FGF-2, BDNF, and vascular endothelial growth factor (VEGF) were induced by antidepressant treatment in several brain regions [9]–[11], and the administration of FGF-2, BDNF, VEGF, and nerve growth factor (NGF) to rodents produced antidepressant-like effects [11]–[14]. Although multiple neurotrophic/growth factors are implicated in antidepressant efficacy [15], the cellular mechanisms for the induction of these factors by antidepressants are unclear.

These neurotrophic/growth factors are produced not only in neurons, but also in astrocytes, which are one of the major glial cells found in the brain [16]. In the human brain, glial cells have been estimated to outnumber neurons in the cerebral cortex, and even in the whole brain, glial cells have contained a similar number of neuronal cells [17]. Although the concept of astrocytes as simply supportive cells for neurons has been accepted for decades, recent studies increasingly support the idea that astrocytes are also excitable cells that play important roles in modulating neuronal plasticity [18], [19]. Furthermore, astrocytes have monoaminergic receptors [20], and regulate the production of neurotrophic/growth factors including FGF-2, BDNF, GDNF and NGF through the activation of monoaminergic receptors [21]–[26]. These findings suggest that astrocytes, as well as neurons, play important roles for the regulation of neurotrophic/growth factors by antidepressants in the brain. In addition, there is increasing evidence indicating that astrocytes are also involved in the pathology of mood disorders [27]. For example, postmortem studies of patients with MDD have revealed a decrease in the density and number of glia in several cortical areas [28], [29], and animal study have shown the pharmacologic ablation of astrocytes in the prefrontal cortex of rats to be sufficient to induce depressive-like behaviors [30].

Treatment with antidepressants affects intracellular signaling, such as the calcium ion and the phosphorylation of mitogen-activated protein kinases in astrocyte cultures [31]–[33]. Our laboratory has revealed that antidepressants induce GDNF secretion through a monoamine-independent mechanism in C6 glioma cells, a model of astrocytes [34]. These reports suggest a novel concept that antidepressants directly act on astrocytes [35]. Therefore, we hypothesized that not only GDNF but also other multiple neurotrophic/growth factors may be directly regulated by antidepressants in astrocytes. We performed the following experiments: First, we investigated the changes in the expression of several neurotrophic/growth factors induced by amitriptyline, a typical tricyclic antidepressant, using astrocyte cultures, in comparison to neuron-enriched cultures, from the cortex where astrocytes dysfunction is assumed in MDD [28], [29]. Second, we compared the effects of amitriptyline and NA on the production of FGF-2 which is abundantly expressed in astrocytes.

## Materials and Methods

### Materials

Amitriptyline, clomipramine, cycloheximide, diazepam, diphenhydramine, fluvoxamine, haloperidol and trihexyphenidyl were obtained from Wako Pure Chemicals (Osaka, Japan); NA, propranolol and 5-HT were from Sigma-Aldrich Co. (St. Louis, MO); duloxetine was from Kenprotec Limited (Middlesbrough, U.K.); prazosin was from Tokyo Chemical Industry (Tokyo, Japan); and yohimbine was from Katayama Chemical Industry (Osaka, Japan).

### Cell Culture

Astrocyte cultures were prepared from neonatal Wistar rats, as described previously [36]. Briefly, the isolated cerebral cortices and hippocampi were minced, and then incubated with trypsin and DNase. Dissociated cells were suspended in Dulbeccco’s Modified Eagle’s Medium (DMEM) supplemented with 10% fetal calf serum and penicillin/streptomycin (100 U/ml and 100 µg/ml, respectively). Thereafter, cell suspensions were plated in 75 cm^2^ tissue culture flasks (8–15×10^6^ cells/flask) precoated with poly-L-lysine (10 µg/ml). The cells were maintained in a 10% CO_2_ incubator at 37°C. After 8–12 days, the cells were purified to remove less adherent neurons and microglia by shaking on a rotary shaker at 100 rpm for 15 h. Adherent cells were trypsinized (0.25%) and plated into 75 cm^2^ flasks. After the cells reached confluence (10 days), the confluent cells were shaken by hand for 10 min. Adherent cells were trypsinized (0.25%) and plated onto new dishes. Using this method, >95% of the cells were detected to express glial fibrillary acidic protein (GFAP), a marker of astrocytes.

Neuron-enriched cultures were also prepared from the cerebral cortex of 1-day-old neonatal Wistar rats. Cell suspensions were prepared as well as the astrocyte cultures, and were plated in 35 mm diameter dishes (3–4×10^6^ cells/dish) precoated with polyethyleneimine (1%). To eliminate proliferative cells, including astrocytes, 10 µM of cytosine β-D-arabino-furanoside hydrochloride (Sigma-Aldrich), a selective inhibitor of DNA synthesis, was added to the mixed cortical cells on the following day. After 6–7 days, the cells were used for experiments. Using this method, >80% of the cells expressed microtubule associated protein-2, a marker of neurons.

All animal procedures were performed in accordance with the Guide for Animal Experimentation, Hiroshima University and the Guideline for Animal Experiments in National Hospital Organization (NHO) Kure Medical Center and Chugoku Cancer Center. The protocols were approved by the Animal Care and Use Committee of Hiroshima University and the Animal Research Ethics Committee, NHO Kure Medical Center and Chugoku Cancer Center.

### RNA Isolation

To collect the total RNA, cells were cultured in serum-free growth medium. After drug treatment, the total RNA was isolated using an RNeasy Mini Kit (Qiagen, Valencia, CA) following the manufacturer’s protocols. The quantity and purity of the RNA were determined with a Multi-Spectrophotometer (Dainippon Sumitomo Pharma Co. Ltd., Osaka, Japan).

### Reverse Transcriptase PCR Analysis

The total RNA was used to synthesize cDNA with MuLV reverse transcriptase (Applied Biosystems, Foster City, CA). PCR assays were performed with the specific primers for the rat adrenergic receptors as described in a previous study [36] and AmpliTag Gold ™ (Applied Biosystems) at 95°C for 10 min followed by 35 cycles of 95°C for 30 s, the respective annealing temperature for 30 s, and 72°C for 2 min, with a final extension at 72°C for 5 min. The resulting PCR products were analyzed on a 1.2% agarose gel and had the sizes expected from the known cDNA sequences.

### Real Time PCR Analysis

The cDNA was synthesized from 500 ng of total RNA using an RNA PCR Kit (avian myeloblastosis virus) version 3.0 (Takara Bioscience, Shiga, Japan). The cDNA was used as a template for real-time PCR. Real-time PCR was performed with the Thermal Cycler Dice® Real Time System II (Takara Bioscience), using TaqMan probes and primers for rat BDNF, the sequences of which were reported previously [37], GDNF, VEGF, FGF-2, NGF, and glyceraldehyde-3-phosphate dehydrogenase (GAPDH) (Applied Biosystems).

### Lactate Dehydrogenase Release Assay

To determine the cytotoxicity of amitriptyline in cortical astrocyte cultures, we measured the lactate dehydrogenase (LDH) release by using a cytotoxicity colorimetric assay kit (Oxford Biomedical Research, Oxford, MI) according to the manufacturer’s instructions. For the LDH release assay, cells were cultured at a density of 0.45×10^6^ cells on a 12 well plate with 1.0 ml of serum-free growth medium. After drug treatment, the conditioned medium was collected and stored at −80°C until being assayed.

### High-performance Liquid Chromatography

The measurement of monoamines (NA, 5-HT and dopamine; DA) and amino acids (glutamate and γ-aminobutyric acid; GABA) was outsourced to SRL (Tokyo, Japan). The concentrations of these molecules in the cultured cells and media were measured by high-performance liquid chromatography (HPLC). The detection limits for NA, 5-HT, DA, glutamate and GABA were 0.03 nM, 4 nM, 0.03 nM, 2 µM, and 2 µM, respectively. The cells and media were collected and stored at −80°C until the outsourcing.

### Immunoblotting Analysis

Cells were maintained in serum-free growth medium. After treatment with various drug concentrations for the indicated incubation times, the cells were solubilized in radioimmunoprecipitation assay buffer with protease inhibitors (100 mM Tris-HCl, pH 7.4, 150 mM NaCl, 1 mM EDTA, 1% Triton X-100, 1% sodium deoxycholate, 0.1% sodium dodecyl sulfate (SDS), 20 µg/ml aprotinin, 20 µg/ml leupeptin, and 1 mM phenylmethylsulfonyl fluoride). Laemli buffer was added to the cell lysates, and they were boiled for 5 min. Equal amounts of protein were separated by 18% SDS-polyacrylamide gel electrophoresis and blotted onto nitrocellulose membranes. The membranes were blocked in blocking buffer for 1.5 h at room temperature and subsequently incubated with a purified polyclonal antibody (pAb) against FGF-2 (1∶1000; Santa Cruz Biotechnology, Santa Cruz, CA), monoclonal antibody against FGF-2 (1∶250; BD Transduction Laboratories, Franklin Lakes, NJ) or β-actin (1∶10000; Sigma-Aldrich) overnight at 4°C. The membranes were washed and then incubated with a horseradish peroxidase–conjugated anti-rabbit or anti-mouse IgG antibody (Santa Cruz Biotechnology) for 1 h at room temperature. Next, the membranes were rinsed, and incubated with Luminescence reagent (Bio-Rad, Tokyo, Japan). Finally, the membranes were exposed to X-ray film to detect the protein. For the quantification of signals, the densities of specific bands were measured with a Science Lab Image Gauge (Fuji Film, Tokyo, Japan). We regularly used anti-FGF-2 pAb (Santa Cruz Biotechnology) in this study, and used another anti-FGF-2 antibody (BD Transduction Laboratories) in order to confirm the presence of three isoforms of the FGF-2 protein.

### FGF-2 Enzyme-linked Immunosorbent Assay

To assess the FGF-2 release, cells were cultured at a density of 0.9–1.2×10^6^ cells on 35 mm diameter dishes with 1 ml of Neurobasal medium (Invitrogen, Carlsbad, CA) supplemented with B27 and L-glutamine. After drug treatment, the conditioned medium was collected and stored at −80°C until being assayed. The levels of FGF-2 release in the conditioned media were determined using a FGF-2 enzyme-linked immunosorbent assay according to the manufacturer’s instructions (R&D Systems, Menneapolis, MN). The minimum detectable concentration of FGF-2 was 0.03 pg/ml.

### Statistical Analysis

We performed the statistical analyses using SPSS software program (SPSS, Chicago, IL). The results are expressed as the means ± SEM. A one-way ANOVA was used in most cases. The differences between the groups were analyzed by the Dunnett's or Tukey honest significant difference (HSD) test. Dunnett’s test was used to compare the control group to the groups treated with the drug. Tukey’s HSD test was used to evaluate whether the observed differences between any two groups were significant. The differences between two groups were analyzed by Student's *t*-test. The significance level was set at p<0.05. The EC_50_ value was calculated using the PRISM software program (GraphPad Software Inc., San Diego, CA).

## Results

### The Effects of Amitriptyline on Neurotrophic/growth Factor mRNA Expression in Astrocyte- and Neuron-enriched Cultures

**Figure 1:**
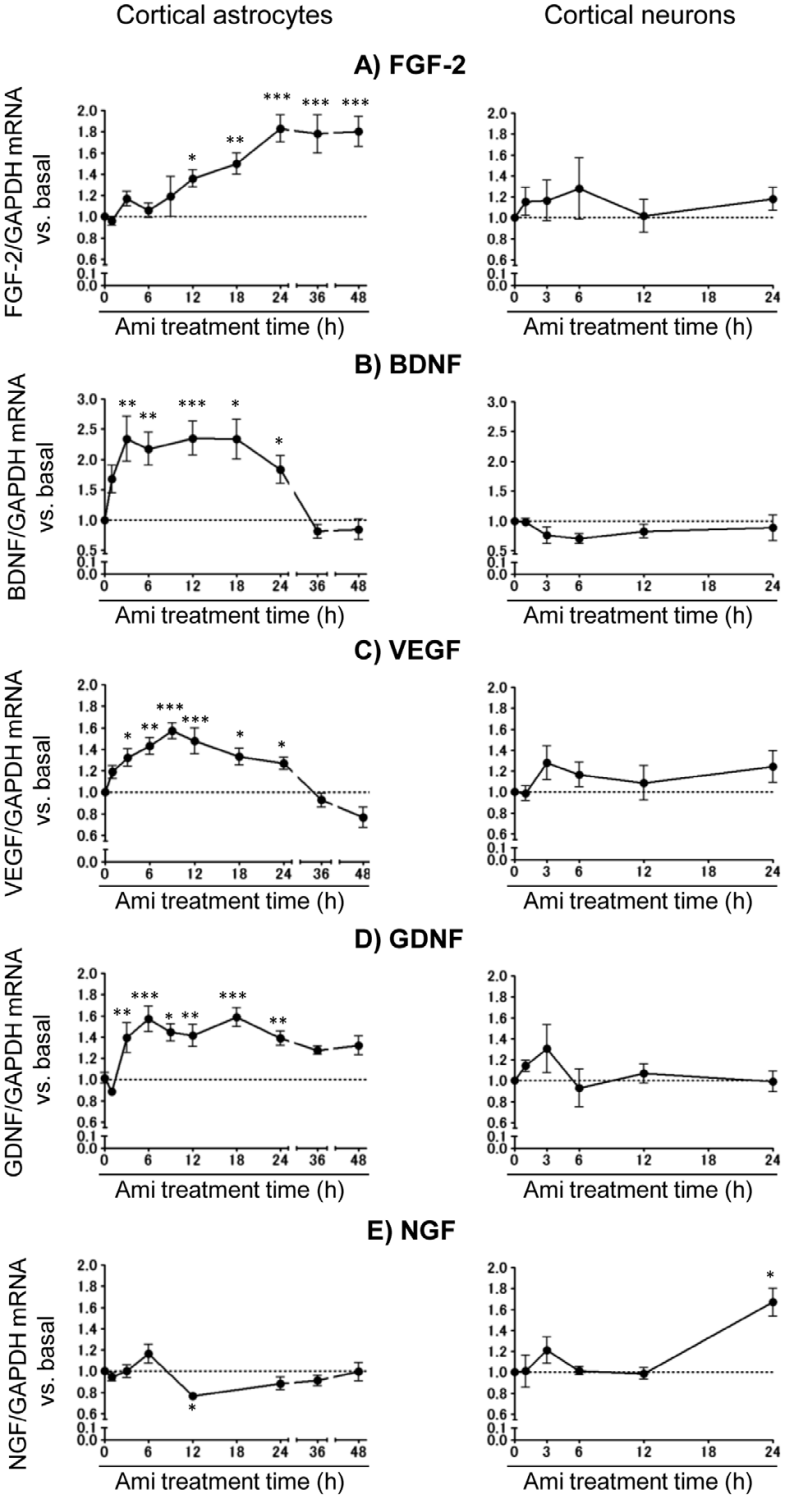
The effects of amitriptyline on the mRNA expression of neurotrophic/growth factors. Cortical astrocyte cultures and neuron-enriched cultures were treated with 25 µM of amitriptyline (Ami) for the indicated periods of time, and the mRNA expression levels of FGF-2 (A), BDNF (B), VEGF (C), GDNF (D), and NGF (E) were analyzed by real time PCR. The values are shown as the ratio of each neurotrophic/growth factor mRNA to GAPDH mRNA. The data are expressed as the means ± S.E.M. *p<0.05, **p<0.01, or ***p<0.001 vs. basal (Dunnett's test; n = 4–11).

We first examined the time course of any changes in the gene expression levels of neurotrophic/growth factors by amitriptyline in cortical astrocyte- and neuron-enriched cultures using real-time PCR. The level of FGF-2 mRNA expression in the astrocyte cultures in response to 25 µM of amitriptyline gradually increased with a peak after 24 h and the increase in FGF-2 mRNA was sustained up to 48 h, while that in the neuron-enriched cultures did not change (Fig. 1A). The expression levels of BDNF, VEGF and GDNF mRNA in the astrocyte cultures rapidly increased with peaks after 3, 6 and 9 h, respectively, and they then returned to the near-baseline levels by 36 h, while those in neuron-enriched cultures did not change (Fig. 1B, C and D). In contrast, the expression level of NGF mRNA transiently decreased in the astrocyte cultures after 12 h and increased in neuron-enriched cultures after 24 h (Fig. 1E).

In order to rule out the possibility that these mRNA changes by amitriptyline were dependent on the duration of the serum-free culture, the total RNA was extracted at the same time points after the drug treatment. In addition, the mRNA expression of GAPDH, as an internal standard, did not significantly change by amitriptyline treatment in astrocyte cultures (amitriptyline 1 h: 1.05±0.05, 6 h: 1.12±0.07, 12 h: 1.06±0.08, 24 h: 0.99±0.05, and 48 h: 0.87±0.06-fold compared to the basal level, respectively) or neuron-enriched cultures (amitriptyline 1 h: 1.14±0.14, 6 h: 1.17±0.14, 12 h: 0.98±0.12, and 24 h: 0.86±0.07-fold compared to the basal level, respectively).

Furthermore, we examined the expression levels of neurotrophic/growth factor mRNA in hippocampal astrocyte cultures that were previously implicated in the pathophysiology of depression [38]. Treatment with 25 µM of amitriptyline for 24 h significantly increased the expression levels of FGF-2, BDNF, and VEGF mRNA, while the expression of GDNF and NGF mRNA remained unchanged (Table 1).

**Table 1:** The effects of amitriptyline on mRNA expression of neurotrophic/growth factors in hippocampal astrocyte cultures.

Factor	mRNA expression (% of basal)
FGF-2	217.0±21.2**
BDNF	269.4±51.0*
VEGF	174.9±17.5**
GDNF	131.2±15.2
NGF	93.7±7.7

Cells were treated with 25 µM of amitriptyline for 24 h, and the mRNA expression levels of FGF-2, BDNF, VEGF, GDNF, and NGF were analyzed by real time PCR. The values are shown as the ratio of neurotrophic/growth factor mRNA to GAPDH mRNA. The data are expressed as the means ± S.E.M. (% of basal) *p<0.05 or **p<0.01 vs. basal (Student's *t*-test; n = 6).

### Comparison of the Expression Levels of Neurotrophic/Growth Factor between Cortical Astrocyte- and Neuron-enriched Cultures

**Figure 2:**
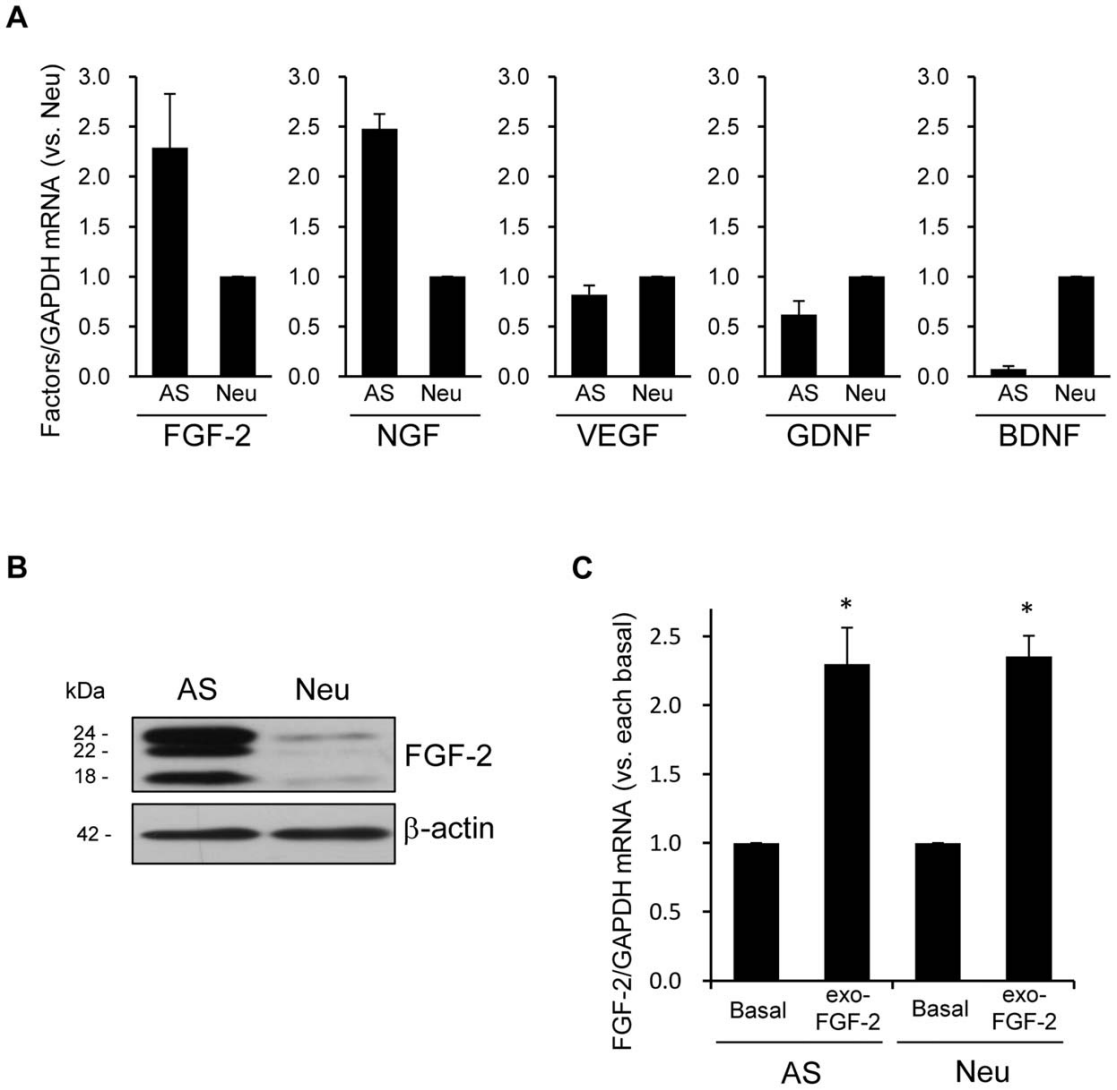
The expression of neurotrophic/growth factors in cortical astrocyte cultures and neuron-enriched cultures. A, The basal mRNA expression levels of neurotrophic/growth factors in astrocyte cultures (AS) and neuron-enriched cultures (Neu). The basal mRNA expression levels of FGF-2, NGF, VEGF, GDNF, and BDNF in AS and Neu were analyzed by real time PCR. The values are shown as the ratio of each factor/GAPDH mRNA level in AS compared with those in Neu. The data are expressed as the means ± S.E.M. B, The basal levels of FGF-2 proteins in AS and Neu. The FGF-2 proteins were detected by an immunoblotting analysis. The β-actin levels were shown as a loading control. Immunoblots from a representative experiment are shown. Similar results were obtained from three independent experiments. C, The effect of exogenous FGF-2 (exo-FGF-2) on FGF-2 mRNA expression in AS and Neu. Each culture was treated with 10 ng/mL of FGF-2 for 3 h, and the FGF-2 mRNA expression was analyzed by real time PCR. The data are expressed as the means ± S.E.M. *p<0.05 vs. each basal (Student's *t*-test; n = 3–4).

We next compared the basal levels of neurotrophic/growth factor mRNA expression between cortical astrocyte- and neuron-enriched cultures. The expression levels of FGF-2 and NGF mRNA in astrocyte cultures were higher than those in neuron-enriched cultures, while the expression levels of VEGF, GDNF, and BDNF mRNA in astrocyte cultures were lower than those of neuron-enriched cultures (Fig. 2A). We focused on the astrocytic FGF-2 expression, which showed a sustained increase in response to amitriptyline and the abundant expression in astrocyte cultures. We further confirmed the comparison of the basal FGF-2 protein levels between cortical astrocyte- and neuron-enriched cultures using immunoblots. FGF-2 protein can be produced as distinct isoforms by alternative initiation of translation of a single mRNA [39]. Thus, the FGF-2 pAb recognized three FGF-2 isoforms (18, 22 and 24 kDa) in rodents. Similar to the results of mRNA levels, all isoforms of the FGF-2 protein in cortical astrocyte cultures were more abundantly expressed than the neurons (Fig. 2B).

Although the cortical neurons obtained from postnatal rats after 6–7 days of *in vitro* culture were relatively young, these neuronal cultures have been known to be functionally-active [40]. We treated the cultures with exogeneous FGF-2 (10 ng/ml, which is a sufficient concentration to induce FGF-2 gene expression in astrocytic and neuronal cultures) as a positive control, in an attempt to confirm whether our cortical neuron-enriched cultures could functionally induce FGF-2 [41], [42]. Treatment with exogeneous FGF-2 for 3 h significantly increased the expression of FGF-2 mRNA to a similar extent (2.5 folds) in both cortical astrocyte- and neuron-enriched cultures (Fig. 2C).

### The Dose-dependent Effect of Amitriptyline on the FGF-2 mRNA Expression in Cortical Astrocyte Cultures

**Figure 3:**
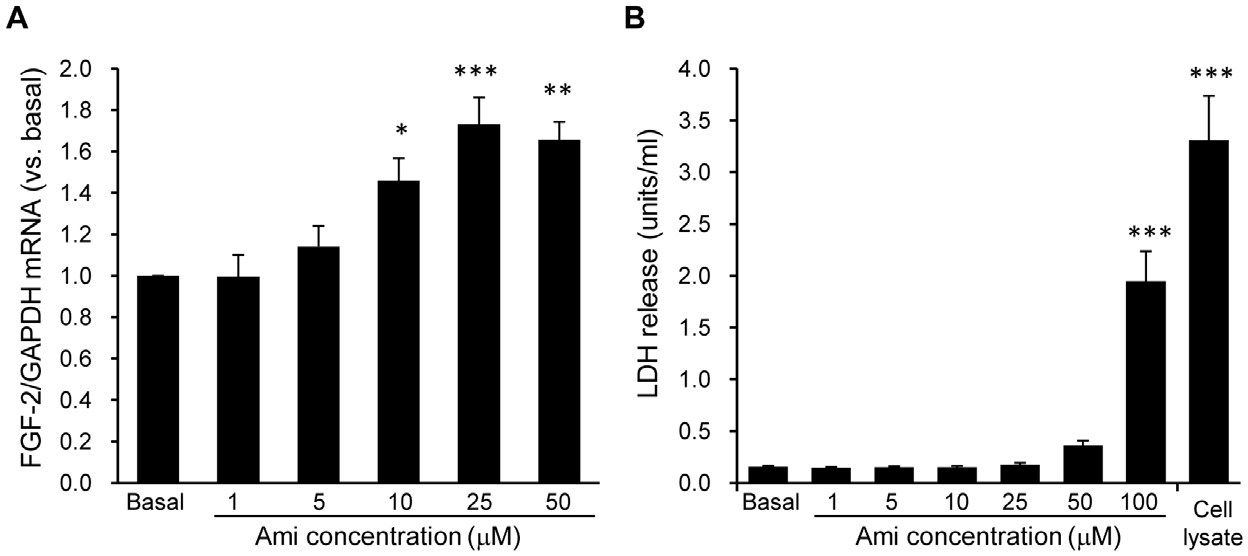
The dose-dependent effect of amitriptyline on FGF-2 expression in cortical astrocyte cultures. A, The dose-dependent increase in FGF-2 mRNA expression in cortical astrocyte cultures induced by amitriptyline (Ami). Cells were treated with the indicated concentrations of Ami for 24 h, and the FGF-2 mRNA expression was analyzed by real time PCR. The values are shown as the ratio of FGF-2 mRNA to GAPDH mRNA. The data are expressed as the means ± S.E.M. *p<0.05, **p<0.01, or ***p<0.001 vs. basal (Dunnett's test; n = 3–6). B, The chemotoxicity of Ami in cortical astrocyte cultures. Cells were treated with the indicated concentrations of Ami for 24 h or were treated with 0.1% of triton X-100 (cell lysate). To determine the cytotoxicity of treatment, we measured the release of lactate dehydrogenase (LDH) in the conditioned media. The data are expressed as the means ± S.E.M. ***p<0.001 vs. basal (Dunnett's test; n = 3–4).

Treatment of cortical astrocyte cultures with amitriptyline for 24 h dose-dependently increased their FGF-2 mRNA expression (Fig. 3A). A statistically significant increase was observed at concentrations above 10 µM, and peaked at 25 µM of amitriptyline. The EC_50_ value was 6.39 µM caluculated by PRISM software. Next, the chemotoxicity of amitriptyline was quantified by a standard measurement of the LDH release. The amount of released LDH remained unchanged after treatment with concentration of 50 µM or less of amitriptyline, and it significantly increased after treatment with 100 µM of amitriptyline and in the whole cell lysates that were used as a positive toxicant control (Fig. 3B). These results showed that treatment with amitriptyline at concentrations of up to 50 µM did not lead to toxicity for the cortical astrocyte cultures. From these results, 25 µM of amitriptyline was used the concentration to obtain the maximum response for FGF-2 mRNA expression in the subsequent experiments.

### The Effects of Amitriptyline on the FGF-2 Protein Levels in Cortical Astrocyte Cultures

**Figure 4:**
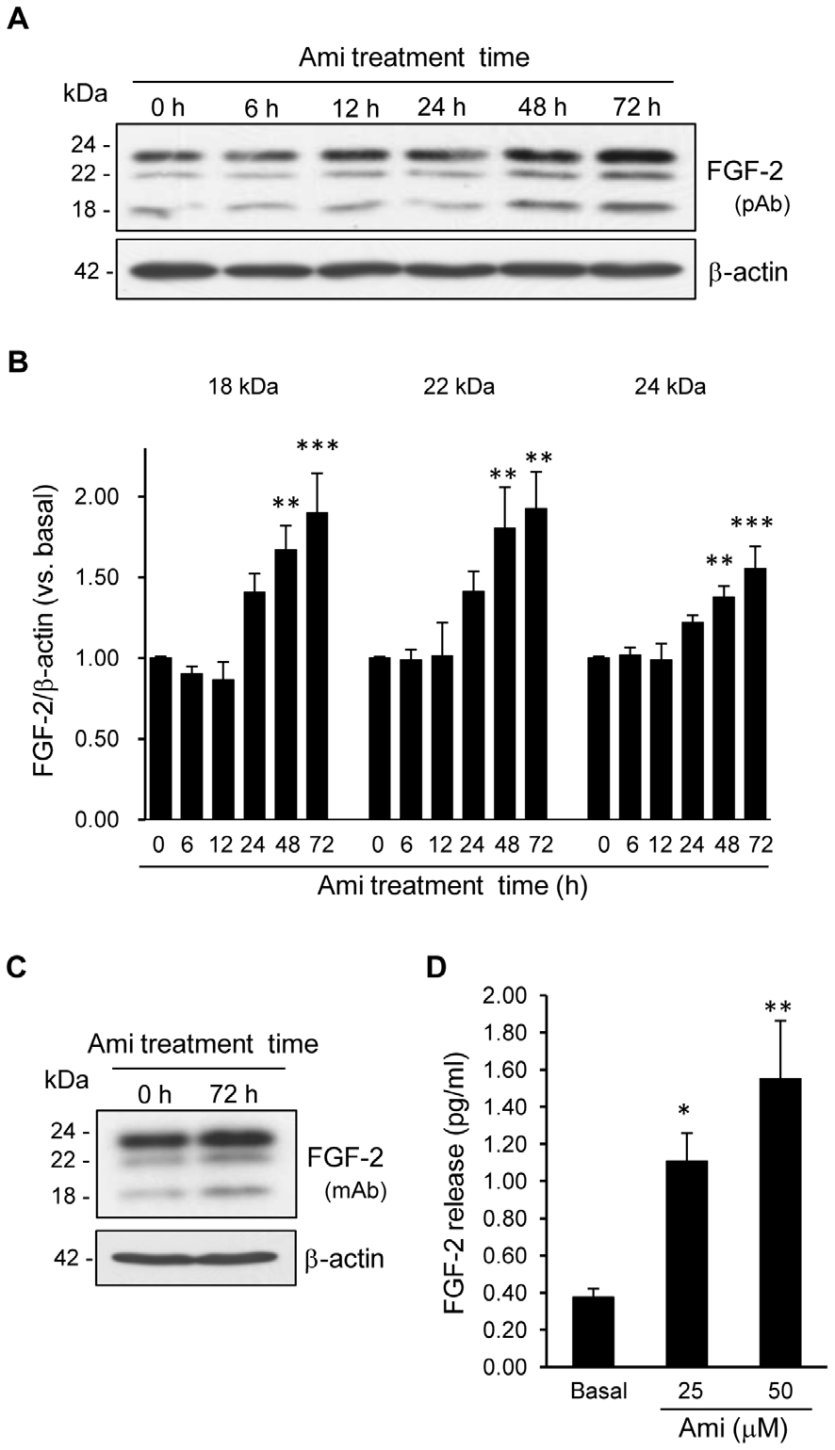
The effect of amitriptyline on FGF-2 production in cortical astrocyte cultures. A, The time course of amitriptyline (Ami)-induced FGF-2 production. Cells were treated with 25 µM of Ami for the indicated periods of time, and the FGF-2 protein levels were analyzed by an immunoblotting analysis using a polyclonal antibody (pAb) against FGF-2 (Santa Cruz Biotechnology). The β-actin levels were used as a loading control. Immunoblots from a representative experiment are shown. B, Quantitation of the protein levels in Fig. A. The values are shown as the ratio of FGF-2 to β-actin. The data are expressed as the means ± S.E.M. *p<0.05, **p<0.01, or ***p<0.001 vs. basal (Dunnett's test; n = 7–13). C, An immunoblotting analysis using another monoclonal antibody (mAb) against FGF-2 (BD Transduction Laboratories). Cells were treated with 25 µM of Ami for 72 h, and the FGF-2 protein levels were analyzed by an immunoblotting analysis. D, The effect of Ami on the FGF-2 release in the conditioned media of cortical astrocyte cultures. Cells were treated with the indicated concentrations of Ami for 48 h, and the FGF-2 release was analyzed by a FGF-2 enzyme-linked immunosorbent assay. The data are expressed as the means ± S.E.M. *p<0.05, or **p<0.01 vs. basal (Dunnett's test; n = 8).

We examined the changes in the FGF-2 protein levels after 6, 12, 24, 48 and 72 h treatment with amitriptyline (25 µM) in the cortical astrocyte cultures. Following the increase in the mRNA expression (12 h treatment), the expression of all three isoforms of the FGF-2 protein significantly increased after 48 and 72 h of treatment with amitriptyline (Fig. 4A and B). Furthermore, we confirmed the increase in all three isoforms of the FGF-2 protein in response to amitriptyline treatment using another anti-FGF-2 antibody (BD Transduction Laboratories) (Fig. 4C). We confirmed that the expression of β-actin as a loading control was not significantly changed by amitriptyline treatment (amitriptyline 24 h: 0.97±0.04, 48 h: 1.03±0.04, and 72 h: 1.09±0.05-fold compared to the basal level, respectively). In addition, we examined the effect of amitriptyline on the FGF-2 release in conditioned medium from cortical astrocyte cultures using ELISA, and observed that treatment with amitriptyline for 48 h dose-dependently increased the FGF-2 release (Fig. 4D).

### Antidepressants Treatment, but not Treatment with Other Psychotropic Drugs, Increased the FGF-2 mRNA Expression in Cortical Astrocyte Cultures

To determine the pharmacological specificity of antidepressants on the FGF-2 mRNA expression, we examined the effects of several different classes of antidepressants and non-antidepressant drugs, including amitriptyline and clomipramine (tricyclic antidepressants), fluvoxamine (a selective serotonin reuptake inhibitor: SSRI), duloxetine (a serotonin noradrenaline reuptake inhibitor: SNRI), haloperidol (an antipsychotic D2-dopamine receptor antagonist), diazepam (a benzodiazepine), diphenhydramine (an antihistaminergic drug), and trihexyphenidyl (an anticholinergic drug). All of the antidepressants significantly increased the FGF-2 mRNA expression in cortical astrocyte cultures, but haloperidol, diazepam, diphenhydramine, and trihexyphenidyl did not (Table 2).

**Table 2:** Antidepressants treatment, but not treatment with other psychotropic drugs, increased the FGF-2 mRNA expression in cortical astrocyte cultures.

Drug	FGF-2 mRNA expression (% of basal)
**Tricyclic antidepressant**	
Amitriptyline	197.9±6.3**
Clomipramine	265.8±13.7***
**Selective serotonin reuptake inhibitor**	
Fluvoxamine	150.6±12.8*
**Serotonin noradrenaline reuptake inhibitor**	
Duloxetine	171.3±14.3**
**Others**	
Diazepam	102.8±8.6
Haloperidol	122.3±13.3
Trihexyphenidyl	115.2±16.6
Diphenhydramine	99.2±13.0

Cells were treated with 25 µM of amitriptyline, clomipramine, fluvoxamine, duloxetine, diazepam, haloperidol, trihexyphenidyl or diphenhydramine for 24 h, and the FGF-2 mRNA expression was analyzed by real time PCR. The values are shown as the ratio of FGF-2 mRNA to GAPDH mRNA. The data are expressed as the means ± S.E.M. (% of basal) *p<0.05, or ***p<0.001 vs. basal (Student's *t*-test; n = 3–4).

### The Amitriptyline-induced Increase in FGF-2 mRNA Expression was Independent of the Monoamine System in Cortical Astrocyte Cultures

**Figure 5:**
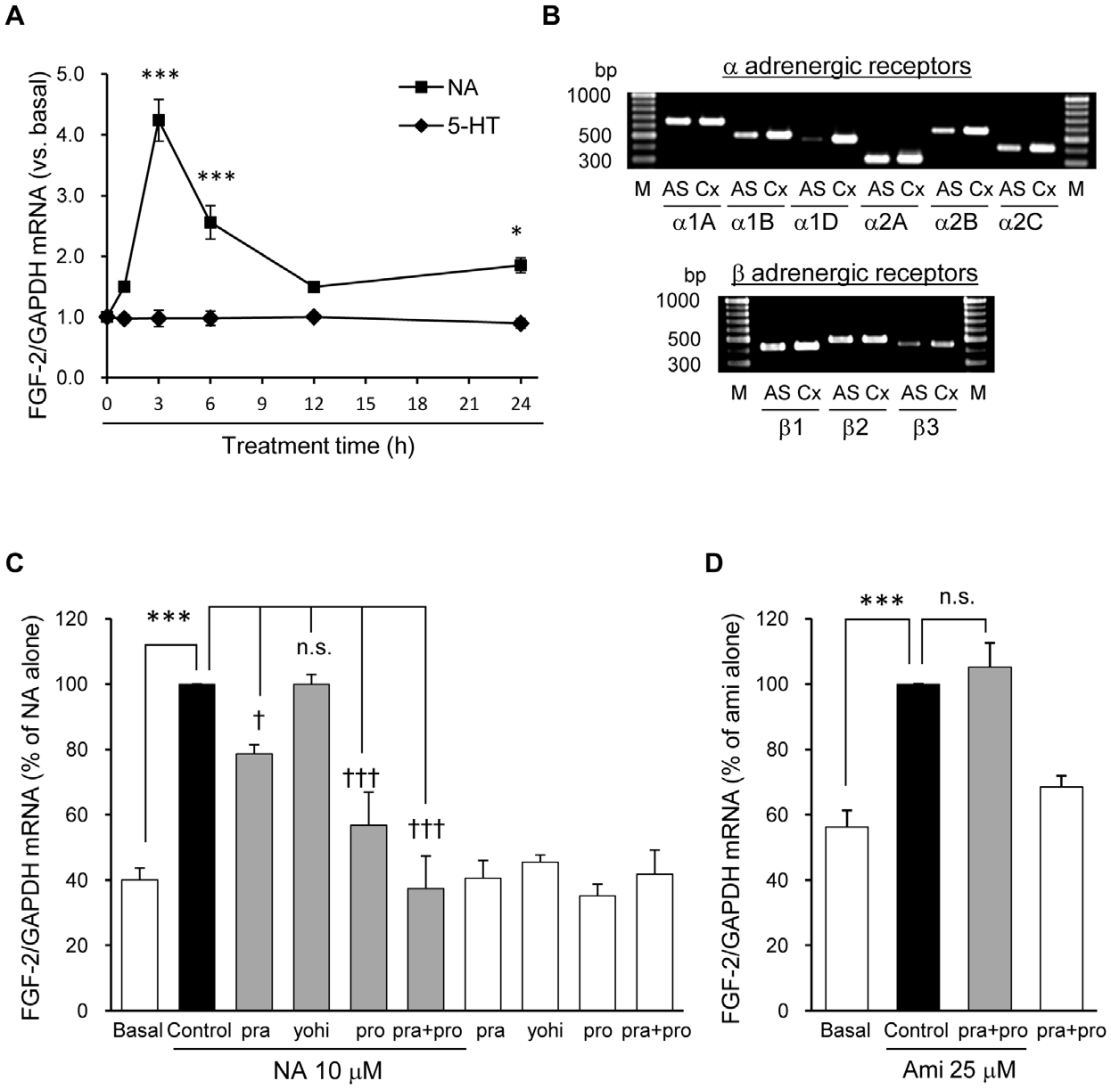
NA, but not amitriptyline, increases the FGF-2 mRNA expression via α1 and β receptors. A, Astrocyte cultures were treated with 10 µM of NA or 5-HT for the indicated periods of time, and the FGF-2 mRNA expression was analyzed by real time PCR. The data are expressed as the means ± S.E.M. **p<0.01, or ***p<0.001 vs. basal (Dunnett's test; n = 3). B, The results of reverse transcriptase PCR analysis of adrenergic receptor mRNA expression. Each lane represented the cDNA fragments of the α1A, α1B, α1D, α2A, α2B, α2C, β1, β2, or β3 adrenergic receptor amplified from the RNA of cortical astrocyte cultures (AS) and the adult rat cortex (Cx). Size markers were shown in Lane M. Cx samples were used as positive controls. C, Astrocyte cultures were pretreated with or without prazosin (pra, 10 µM), yohimbine (yoh, 10 µM) or propranolol (pro, 10 µM) for 30 min,and treated with NA (10 µM) for 3 h. The FGF-2 mRNA expression was analyzed by real time PCR. The data are expressed as the means ± S.E.M. (% of NA alone) ***p<0.001 vs. basal, n.s.; not significantly different from the control (NA only) group, and †*p*<0.05 or †††*p*<0.001 vs. NA alone (Tukey’s HSD test; n = 3–4). D, Astrocyte cultures were pretreated with or without both pra (10 µM) and pro (10 µM) for 30 min and treated with amitriptyline (Ami, 25 µM) for 24 h. The FGF-2 mRNA expression was analyzed by real time PCR. The data are expressed as the means ± S.E.M. (% of Ami alone) ***p<0.001 vs. basal and n.s.; not significantly different from Ami alone (Tukey’s HSD test; n = 3).

Antidepressants are known to inhibit monoamine (NA and/or 5-HT) transporters or monoamine oxidase and to increase the monoamine levels in the extracellular space. In addition, NA increases the expression of FGF-2 in rat astrocyte cultures [21]. Therefore, we treated the astrocyte cultures with a high concentration of NA and 5-HT (10 µM) to clarify whether monoamines affect the expression of FGF-2 mRNA in our cortical astrocyte cultures. Treatment with NA significantly increased the expression of FGF-2 mRNA, which reached a maximum at 3 h. On the other hand, treatment with 5-HT had no effect on the expression of FGF-2 mRNA (Fig. 5A). Next, we examined which subtypes of adrenergic receptors might play a role in the effects of NA on the FGF-2 mRNA expression. As shown in Fig. 5B, the expression of all adrenergic receptors was detected in cortical astrocyte cultures by the reverse transcriptase PCR analysis. As shown in Fig. 5C, pretreatment with either prazosin (a selective α1-adrenergic receptor antagonist) or propranolol (a nonselective β-adrenergic receptor antagonist) partially blocked the NA-induced FGF-2 mRNA expression. In addition, co-treatment with both antagonists completely reversed the effects of NA on the expression of FGF-2 mRNA. In contrast, preincubation with yohimbine (a selective α2-adrenergic receptor antagonist) had no influence on the effect of NA. These results showed that both α1 and β receptors were involved in the action of NA on the cortical astrocytes.

We examined the effects of prazosin and propranolol on the amitriptyline-induced increase in FGF-2 mRNA expression, to clarify the role of adrenergic receptors in the antidepressant-induced increase in FGF-2 expression in astrocyte cultures. Co-incubation with both prazosin and propranolol did not affect the amitriptyline-induced change in FGF-2 mRNA expression (Fig. 5D). These results suggest that amitriptyline induces FGF-2 mRNA expression in an adrenergic receptor-independent manner.

To eliminate the possibility that NA is involved in the effect of amitriptyline in astrocyte cultures, we analyzed the NA concentration in the cell lysates and conditioned media from astrocyte cultures incubated with or without 25 µM of amitriptyline for 24 h. However, no detectable NA was present in either the cell lysates or the conditioned media as determined by HPLC. The detection limit for NA was 0.03 nM. Likewise, 5-HT and DA were not detectable in either the cell lysates or the conditioned media.

### Cycloheximide Differentially Affected the Amitriptyline- or NA-induced Increase in FGF-2 Expression in Cortical Astrocyte Cultures

**Figure 6:**
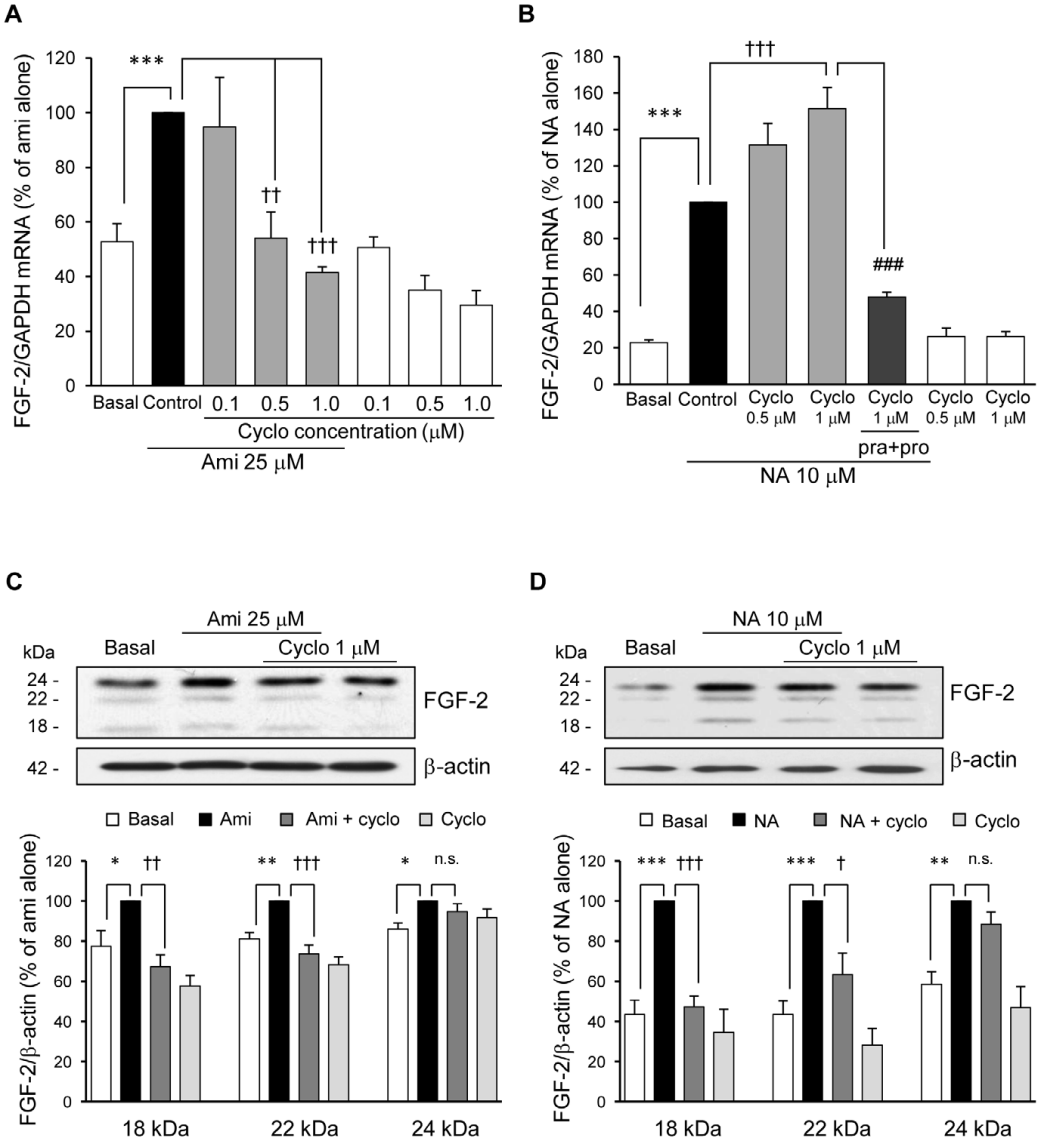
Amitriptyline, but not NA, induces FGF-2 expression through *de novo* synthesis in astrocyte cultures. A, Cells were pretreated with or without cycloheximide (Cyclo) for 10 min and treated with amitriptyline (Ami, 25 µM) for 24 h. The FGF-2 mRNA expression was analyzed by real time PCR. The data are expressed as the means ± S.E.M. (% of Ami alone) *p<0.05 vs. basal, and †*p*<0.05 or ††*p*<0.01 vs. Ami alone (Tukey’s HSD test; n = 3). B, Cells were pretreated with or without Cyclo for 10 min or prazosin (pra, 10 µM) and propranolol (pro, 10 µM) for 30 min and treated with NA (10 µM) for 3 h. The FGF-2 mRNA expression was analyzed by real time PCR. The data are expressed as the means ± S.E.M. (% of NA alone) ***p<0.001 vs. basal, †††*p*<0.001 vs. NA alone, and ###*p*<0.001 vs. NA and Cyclo (1 µM) (Tukey’s HSD test; n = 3). C, Cells were pretreated with or without Cyclo (1 µM) for 10 min and treated with 25 µM of Ami for 72 h. The FGF-2 protein levels were analyzed by an immunoblotting analysis. The data are expressed as the means ± S.E.M. (% of Ami alone) *p<0.05 or **p<0.01 vs. basal, and ††*p*<0.01 or †††*p*<0.001 vs. Ami alone (Tukey’s HSD test; n = 8). D, Cells were pretreated with or without Cyclo (1 µM) for 10 min and treated with 10 µM of NA for 24 h. The FGF-2 protein levels were analyzed by an immunoblotting analysis. The data are expressed as the means ± S.E.M. (% of NA alone) **p<0.01 or ***p<0.001 vs. basal, and †*p*<0.05 or †††*p*<0.001 vs. NA alone (Tukey’s HSD test; n = 4–5).

Because the increase of FGF-2 mRNA expression by amitriptyline occurred relatively later (24 h) than that of other factors (Fig. 1), we investigated whether amitriptyline induces FGF-2 expression via *de novo* protein synthesis using cycloheximide, a general inhibitor of protein synthesis. Pretreatment of cortical astrocyte cultures with the indicated concentrations of cycloheximide dose-dependently and completely blocked the amitriptyline-induced increase in FGF-2 mRNA expression (Fig. 6A). A concentration of cycloheximide (1 µM) has previously been used to adequately inhibit protein synthesis in astrocyte cultures [43]. We also examined the effect of cycloheximide on the NA-induced increase in FGF-2 expression. In contrast to amitriptyline, pretreatment with 1 µM of cycloheximide enhanced the NA-induced increase in FGF-2 mRNA expression. This enhancing effect of cycloheximide was blocked by prazosin and propranolol pretreatment (Fig. 6B). These results suggest that this cycloheximide-induced effect was mediated via the α1 and β-adrenergic receptors. These results further supported that the mechanism underlying the amitriptyline-induced increase in FGF-2 was distinct from that of NA. We further confirmed the effects of cycloheximide on the amitriptyline- or NA-induced FGF-2 protein levels. Pretreatment with cycloheximide blocked both the amitriptyline- and NA-induced 18 and 22 kDa isoforms, but not the 24 kDa isoform (Fig. 6C and D). The 24 kDa isoform was abundantly expressed in this culture, and has been reported to localize more in the nucleus compared with the other low molecular weight isoforms [39]. This isoform may not be as susceptible to the effect of cycloheximide as other isoforms, probably because there is less degradation or turnover of protein [44].

## Discussion

Several conclusions can be drawn from the present study. First, the treatment of cortical astrocyte cultures, but not neuron-enriched cultures with amitriptyline increased the expression of multiple neurotrophic/growth factors including FGF-2, BDNF, GDNF, and VEGF, all of which have been implicated in the effect of antidepressants. The increases in most factors were observed in astrocyte cultures from the hippocampus, which has been also implicated in the efficacy of antidepressant. Second, the increase in FGF-2 mRNA expression in astrocyte cultures was selectively induced by several different types of antidepressants. These results suggest the possible specificity of this effect of antidepressants on astrocytes. Third, amitriptyline and NA had different inductive mechanisms on the FGF-2 production in astrocyte cultures.

Our results demonstrated that treatment with amitriptyline increased the expression of the four antidepressant effect-related neurotrophic/growth factors with a different time course in rat cortical astrocyte cultures. Different types of antidepressants also increased the expression of FGF-2 in astrocyte cultures. Previous *in vitro* studies have reported that antidepressants affect the expression of neurotrophic/growth factor in astrocytes. For example, treatment with fluoxetine, a SSRI, up-regulated BDNF, GDNF and/or VEGF mRNA expression in rodent cortical astrocyte cultures [33], [45]. In addition, an *in vivo* study reported that antidepressants affect astrocytes in rat brain. For example, the reduction of the astrocytic marker GFAP in the rat hippocampus after chronic unpredictable stress is reversed by the tricyclic antidepressant, clomipramine, accompanied by the improvement of depressive-like behaviors [46]. These results led us to hypothesize that astrocytes might be one of the targets of antidepressants, thus leading to the production of several neurotrophic/growth factors.

There were differences in the expression of neurotrophic/growth factors in response to amitriptyline in the cortical and hippocampal astrocyte cultures (Fig. 1 and Table 1). In particular, the expression of GDNF mRNA did not significantly increase after the administration of amitriptyline in the hippocampal astrocyte cultures. A previous report showed the basal hippocampal GDNF mRNA levels to be lower than the cortical levels due to differential DNA methylation at GDNF promoter sites in mice [47]. Therefore, the inconsistent GDNF mRNA expression derived from differential brain regions regulated by amitriptyline might be due to a difference in the epigenetic states in different brain regions. In addition, the primary cultures of astrocytes contain few cells that retain the properties of neuronal stem cells [48]. Another possibility is that cortical and hippocampal astrocyte cultures might be composed of different proportions of stem/progenitor cells.

This study demonstrated that, in comparison to amitriptyline, NA acutely and transiently increased the FGF-2 mRNA expression in cortical astrocyte cultures. In addition, the NA-induced increase in FGF-2 mRNA expression was mediated the α1 and β-adrenergic receptors, while the amitriptyline-induced increase in FGF-2 mRNA expression was independent of the adrenergic receptors. Furthermore, pretreatment with cycloheximide showed an opposing effect on amitriptyline- and NA-induced FGF-2 mRNA expression in cortical astrocyte cultures. These results indicated that amitriptyline increased the FGF-2 mRNA expression via a pathway distinct from NA-induced expression in the astrocytes. Moreover, monoamines (NA, 5-HT and DA) were not detected by HPLC in our cortical astrocyte cultures. Therefore, our findings suggest that amitriptyline may have a novel mechanism for inducing FGF-2 through a monoamine-independent pathway in the astrocytes.

Several lines of evidence have demonstrated the relevance of FGF-2 to depression. Indeed, the expression of FGF-2 is down-regulated in postmortem brains of depressed patients [5], and increased by antidepressants in rat several brain regions [10], [49]. Furthermore, intracerebroventricular administration of FGF-2 produces antidepressant-like effects in different types of animal models of depression [13], [50], [51]. In addition, antidepressant efficacy is blocked in FGF-2 deficient mice in the tail suspension test [50], and blocked by treatment with a FGF receptors (FGFR) inhibitor in the chronic unpredictable stress model of depression [51]. These reports strongly indicated that FGF-2 signaling mediates the therapeutic actions of antidepressants. Our results demonstrated that astrocytic FGF-2 production was induced in a monoamine-dependent (via NA) and -independent (via amitriptyline) manner. Therefore, direct action on astrocytes might play a role in the therapeutic effects of antidepressants through the FGF-2 production in cooperation with the activation of monoamine transporters by antidepressants.

Our results demonstrated that treatment with amitriptyline increased the secretion of FGF-2 proteins in a dose-dependent manner. Figure 2C shows that exogenous FGF-2 treatment increased the intrinsic FGF-2 mRNA expression both in astrocyte and neuron-enriched cultures. These results suggest that FGF-2 secreted from astrocytes might affect the neighboring astrocytes and neurons through the FGF-2 signaling in the brain. The FGF-2 could influence neuronal plasticity via neurogenesis and synaptogenesis [52], [53]. Indeed, even low concentration levels of FGF-2 (0.2–2 pg/ml), seen in the astrocyte cultures in this experiment, promotes neurogenesis in neural progenitor cell cultures [54]. Further studies are needed to confirm the involvement of astrocytic FGF-2 induced by antidepressants in neurotrophic function using a neuron-astrocyte co-culture system and utilizing animals, including those with the conditional knock-down of astrocytes.

We investigated the effect of cycloheximide on the amitriptyline-induced FGF-2 mRNA expression in order to determine the mechanism that causes the amitriptyline-induced increase in FGF-2 mRNA to occur later than that of other factors in cortical astrocyte cultures. As a result, treatment with cycloheximide blocked the increase in amitriptyline-induced FGF-2 mRNA expression. Thus, certain factors produced via the *de novo* protein synthesis following amitriptyline treatment seem to be involved in the regulation of FGF-2 mRNA in astrocyte cultures. Possible candidates include, 1) neurotrophic/growth factors which are induced by amitriptyline earlier than FGF-2, and 2) intracellular signaling molecules, such as transcription factors which induce FGF-2 mRNA expression. Treatment with BDNF, VEGF or GDNF (10 ng/ml) for 3 h did not increase the FGF-2 mRNA expression in cortical astrocyte cultures (data not shown). Therefore, it appears that neurotrophic/growth factors which are induced by amitriptyline earlier than FGF-2 are unlikely to be the candidate molecules. Previous studies have indicated that the cAMP/PKA-signaling pathway, early growth response-1, or myeloid zinc finger protein 1 activate FGF-2 transcription in human or rat astrocyte cultures [42], [55], [56]. Therefore, these molecules might be candidates involved in the effect of amitriptyline. Further investigations are needed to clarify the candidate molecules. On the other hand, the NA-induced increase in FGF-2 mRNA expression was not blocked, but was rather enhanced, by cycloheximide. This result closely correlates with the previous findings [21] which showed that the increase of FGF-2 mRNA expression by isoproterenol (a nonselective β-adrenergic receptor agonist) had a tendency to be enhanced by cycloheximide in astrocyte cultures. Cycloheximide may enhance the NA-induced increase in FGF-2 mRNA by several mechanisms, such as the inhibition of the synthesis of an mRNA destabilizing factor, or by the inhibition of the synthesis of a transcriptional inhibitor.

We have previously reported that amitriptyline activated FGFR through the matrix metalloproteinase (MMP)-dependent shedding of FGFR ligands in a monoamine-independent manner in C6 cells [57]. Therefore, the activation of FGFR through the MMP might be involved in the amitriptyline-induced increase in FGF-2 expression in astrocytes. We are currently performing further studies utilizing pharmacological inhibition of FGFR and silencing the gene expression of FGFR to uncover the involvement of FGFR signaling on the amitriptyline-induced increase in FGF-2 expression.

In this study, we treated astrocyte cultures with antidepressants at higher concentrations than are clinically relevant with regard to the therapeutic plasma level, but the LDH release assay showed that a concentration of 50 µM or less of amitriptyline was not cytotoxic. Furthermore, most antidepressants have been reported to accumulate in the brain because of their highly lipophilic properties [58]. For example, the brain concentration of amitriptyline is approximately 10 to 35 times higher than the corresponding blood levels [59]–[61]. The therapeutic plasma concentrations of amitriptyline range from approximately 0.36 to 0.9 µM [62]. Therefore, these findings suggest that amitriptyline may regulate the neurotrophic/growth factor expression in the astrocytes at clinically relevant brain concentrations.

We found that the treatment of the cortical neuron-enriched cultures with amitriptyline did not induce FGF-2 and BDNF mRNA expression. However, previous *in vivo* studies have shown that antidepressants increased the expression of FGF-2 and BDNF in the neurons of cortex or hippocampus of the rat brain [9], [10]. This discrepancy could be explained as follows: In the neuron-enriched cultures obtained from neonatal rat cortex in this study, monoamines were not detected, while glutamate and GABA were detected by HPLC (data not shown). These results suggest that the cortical neuron-enriched cultures are mainly included the glutamatergic or GABAnergic neurons. Our preliminary studies have demonstrated that NA increased the expression of FGF-2 in neuron-enriched cultures (unpublished observations), and a previous report showed that NA increased the expression of BDNF in rat neuronal cultures [63]. Therefore, because the neuron-enriched cultures in this study hardly included any monoaminergic neurons, amitriptyline was not observed to induce FGF-2 and BDNF mRNA expression.

In conclusion, multiple neurotrophic/growth factor systems seem to be cooperatively involved in the therapeutic effect of antidepressants in the brain. Especially, antidepressant-induced FGF-2 production might participate in the direct effect of antidepressants on astrocytes in a monoamine-independent manner. Clarifying the monoamine-independent novel targets of antidepressants in astrocytes might contribute to the development of more efficient treatments for MDD.
